# Ontogenetic Analysis of *Chelonus formosanus* and Diversity of Its Internal Microbiota

**DOI:** 10.3390/insects16020180

**Published:** 2025-02-08

**Authors:** Jingjing Jia, Qing Feng, Weikang Huang, Zhufeng Lin, Xuncong Ji

**Affiliations:** 1 Institute of Plant Protection, Hainan Academy of Agricultural Sciences, Haikou 571100, China; j9405136318@163.com (J.J.);; 2 Research Center of Quality Safety and Standards for Agro-Products, Hainan Academy of Agricultural Sciences, Haikou 571100, China; 3 Hainan Key Laboratory for Control of Plant Diseases and Insect Pests, Haikou 571100, China

**Keywords:** *Chelonus formosanus*, morphology, 16S rDNA sequencing, microbiota

## Abstract

*Chelonus formosanus* is a parasitic wasp capable of parasitizing various Noctuidae pests, including the highly invasive *Spodoptera frugiperda*, and it demonstrates strong pest control potential. Both egg and larval stages primarily occur within the host organism, and the total developmental time from egg to adult is approximately 19.62 days. To investigate the microbial communities at different stages, we performed 16S rDNA sequencing (V1–V9 region) using PacBio sequencing and identified 404 bacterial species belonging to 61 classes, 116 orders, 182 families, and 308 genera across larval, pupal, female, and male adult stages. Bacterial diversity and richness varied across the stages, with *Enterobacter* and *Enterococcus* dominating in larvae and pupae and *Pseudomonas* emerging as dominant in female adults. In contrast, male adults predominantly resided with *Ralstonia* and *Achromobacter*. The predicted functions of bacteria within *C. formosanus* at different developmental stages are predominantly marked by the high abundance of metabolic pathways. This study provides a comprehensive understanding of the morphology of *C. formosanus* and contributes to the practical control of host pests. Additionally, our findings preliminarily characterized the microbial community of various developmental stages, laying the groundwork for its functional study.

## Simple Summary

*Chelonus formosanus* (Sonan) is an egg–larval parasitoid wasp with enormous potential for the biological control of various Noctuidae pests. However, little is known about its ontogenetic development and the associated microbiota. In this study, we examined the ontogenetic characteristics of *C. formosanus*. We performed 16S bacterial amplicon sequencing at different developmental stages. This study elucidates the morphological and developmental dynamics of *C. formosanus* and reveals its microbiota abundance at different developmental stages. Our findings lay a solid foundation for understanding the complexity of core microorganisms colonizing *C. formosanus*.

## 1. Introduction

*Chelonus formosanus* (Sonan) (Hymenoptera: Braconidae) is an egg–larval parasitoid wasp and serves as a natural enemy to various pest species within the Noctuidae family [1]. Recent reports have highlighted its role as a significant biological control agent against *Spodoptera frugiperda*, a globally invasive pest, in regions such as China [2], India [3], the Philippines [4], and Japan. Notably, in New Delhi, *C. formosanus* is the dominant parasitic wasp attacking *S. frugiperda* [5]. Parasitism rates of *C. formosanus* on *S. frugiperda* eggs ranged from 6.8% to 17.65% under natural conditions [2,4]. Moreover, large-scale rearing of *C. formosanus* is feasible when *S. frugiperda* is used as a host [3]. The highest parasitism rate of *C. formosanus* on *S. frugiperda* eggs could reach 100% under both indoor and field cage conditions, demonstrating its considerable potential for biocontrol. Although previous studies have primarily revealed the morphological traits of *C. formosanus* adults, there is a dearth of information regarding its developmental stages, particularly the larval and pupal phases.

Insects host a diverse and abundant array of microorganisms, including bacteria, archaea, and eukaryotes. These microbes are widely distributed across various insect tissues, including the body wall, intestine, hemolymph, and cells [6,7,8]. Recent advancements in sequencing technologies have significantly enhanced the study of insect-associated microbiota, drawing increasing attention from the scientific community. Although insects are associated with a variety of microbes, bacteria are the most widespread and common category. The impact of bacteria on insect physiology is multifaceted. For example, bacterial communities can promote the growth and development of larvae in species such as *Bactrocera dorsalis* and *Plagiodera versicora* [9,10], motivate the foraging decision and promote fecundity and survival of *B. dorsalis* [11], alter oviposition preferences of *Encarsia pergandiella* [12], and protect hosts from pathogenic threats and environmental stressors [6]. Furthermore, bacterial supplementation has been shown to optimize body weight gain in *Hermetia illucens* larvae with reduced food intake [13] and aid poikilothermic insects in adapting to climate change by boosting host immune responses [14].

Microbial diversity in insects is shaped by various factors, including species, diet, and geographic origin. Previous research has shown that the number of symbiotic bacteria in the four *Sclerodermus* parasitoids did not differ significantly; however, the composition of their bacterial communities was significantly different [15]. In other insects, such as *Grapholita molesta* [16], *Harmonia axyridis* [17], and *S. frugiperda* [18], the diversity and composition of microbial flora was affected by host species. For instance, the bacterial diversity and richness index in three different *Rhyzopertha dominica* populations sampled from different grain storage locations were significantly higher compared to the populations from laboratory settings [19]. Additionally, bacterial community composition in *Diaphorina citri* exhibited notable geographic variation [20]. However, some insects show minimal sensitivity to distinct diets and geographic locations and no significant differences were found in the gut microbiota diversity of *Apriona germari* larvae collected from *Populus tomentosa* and *Malus pumila* [21]. In the case of *Riptortus pedestris*, the gut microbiome composition of 12 populations across distinct geographic regions was largely defined by geographic or soil factors [22]. Furthermore, no significant variation in microbial richness and diversity was observed in *Chironomus circumdatus* sampled at three different locations [23].

Throughout the life cycle of insects, the composition of symbiotic bacterial communities undergoes dynamic changes. These changes may involve the loss, recombination, or acquisition of new symbiotic bacteria, with such fluctuations being most pronounced in holometabolous insects [24]. For example, the bacterial composition in *Brithys crini* shows significant variation among different developmental stages [25]. Similarly, the gut microbiomes of dragonfly nymphs differ from those of adults [26], and *Chironomus transvaalensis* exhibits a reduction in bacterial diversity with its development from egg to adult [27]. There are remarkable differences in the gut bacteria community between different instar larvae and diapause prepupae of *Colleles gigas*. The bacterial diversity decreases gradually from early instar to late instar and then to diapause prepupa [28]. Moreover, the bacterial abundance and diversity in *Propylaea japonica* and *Osmia excavata* also vary among different developmental stages [29,30]. However, some studies have found that the gut microbiome of *Aldrichina grahami* is not significantly influenced by diet or developmental stage [31]. To date, there are no reports relevant to the ontogenetic process and associated bacterial communities of *C. formosanus* at different developmental stages. Given the crucial role of bacteria in insect physiology, understanding the microbial dynamics of *C. formosanus* is essential for its potential utilization in controlling Noctuidae pests.

To gain a deeper understanding of *C. formosanus* biological characteristics and deploy itin practical pest management, it is crucial to investigate the developmental characteristics of *C. formosanus* within the host. This study aims to fill this knowledge gap, thereby providing a solid theoretical foundation for the enhanced use of *C. formosanus* in the control of *S. frugiperda*. This study aims to investigate the ontogenetic development of *C. formosanus* using ultra-depth-of-field three-dimensional microscopy and to analyze its microbiota community at various developmental stages by 16S rDNA sequencing. Clarifying the morphological and developmental changes of *C. formosanus* and characterizing the bacterial species and their dynamics at different stages will lay a solid foundation for further investigation into its role in modulating the growth, reproduction, and parasitism of host species.

## 2. Materials and Methods

### 2.1. Insect Rearing

The *S. frugiperda* and *C. formosanus* colonies used in this study were collected from Daoyu Village, Xiuying District, Haikou City, Hainan Province, China (19°52′22.47″ N, 110°9′56.48″ E). The specimens were transported to the laboratory for multi-generational breeding and are currently maintained at the Institute of Plant Protection, Hainan Academy of Agricultural Sciences. *S. frugiperda* was reared on maize leaves, while *C. formosanus* was maintained on *S. frugiperda* eggs for over 30 generations. The insects were housed in an intelligent artificial climate chamber with a temperature of 26 ± 1 °C, a relative humidity of 70 ± 10%, and a photoperiod of 12 h of light and 12 h of darkness (SAFE-RGQHS-2, Ningbo Saifu experimental instrument Co., Ltd., Ningbo, China). Additionally, the insects were provided with a nutritional supplement consisting of 10% honey water.

### 2.2. Experimental Method

#### 2.2.1. Determination of Individual Development of *Chelonus formosanus*

Fresh eggs of *S. frugiperda* were collected and subsequently exposed to mated female *C. formosanus* adults in a sealed rearing apparatus. After being parasitized by *C. formosanus,* the parasitized *S. frugiperda* eggs were immediately removed and dissected to examine the initial oviposition of *C. formosanus*. Additional eggs were kept for subsequent observations, with the host being dissected every 24 h. The developmental stages of parasitic wasps were visualized using a Keyence VHX-7000 digital microscope, and the length was measured (eggs and larvae aged from one to seven days were preserved in PBS for imaging), and 30 larvae were dissected per session. The average developmental stage of multiple replicated measurements was recorded to represent the wasp’s developmental status at each time point. The wasp’s developmental duration was monitored at different stages, and each measurement was repeated 100 times.

#### 2.2.2. Samples Collection

The larvae (L) were collected 10 days after parasitization and development in the host body, while the pupae (P) were collected on the first day of pupation. Newly emerged, unmated female (AF) and male (AM) adults were also collected accordingly. After disinfecting the surfaces of the collected *S. frugiperda* larvae and *C. formosanus* cocoons, the surfaces were opened using tweezers to extract larvae and pupae of *C. formosanus*. Then, all specimens were disinfected with 75% ethanol for three minutes under a laminar flow hood, followed by three washes with PBS buffer (pH 7.2–7.4) each for one minute. After washing, the specimens were immediately frozen in liquid nitrogen and stored at –80 °C. The sample sizes were as follows: 50 larvae, 30 pupae, 20 female adults, and 20 male adults per treatment group. Each treatment was repeated three times.

#### 2.2.3. DNA Extraction and 16S rDNA Sequencing

Total DNA was extracted using the TGuide S96 magnetic bead DNA extraction kit (DP812, Tiangen Biotech Co., Ltd., Beijing, China). The DNA concentration was measured by combining 1X dsDNA HS Working Solution with an enzyme labeler for detection. Subsequently, detection and amplification were carried out on a Bailing 1000 automated platform (Revvity Biomedical Co., Ltd., Shanghai, China), depending on the determined DNA concentration and the target amplification region. The V1–V9 region of the 16S rRNA gene was amplified using the primers 27F (16S-F) (5′-AGRGTTTGATYNTGGCTCAG-3′) and 1492R (16S-R) (5′-TASGGHTACCTTGTTASGACTT-3′). The PCR master mix consisted of 2 µL genomic DNA, 6.5 µL NFW, 10 µL KOD ONE MM, and 1.5 µL of barcode primer pairs. The amplification was as follows: pre-denaturation at 95 °C for 2 min, denaturation at 98 °C for 10 s, annealing at 55 °C for 30 s, extension at 72 °C for one minute and 30 s, followed by 22 cycles. A final extension was carried out at 72 °C for two minutes. The integrity of the PCR products was assessed by electrophoresis on a 1.8% agarose gel. For library construction of the amplified products, the SMRTBELLS PREP KIT 3.0 (PacBio, Menlo Park, CA, USA) was used for damage repair, terminal repair, and adapter ligation of the amplified products. The reaction was carried out on a PCR instrument, and the resulting library was purified using AMpure PB magnetic beads (Beckman Coulter Trading Co., Ltd., Shanghai, China). The purified library was quantified using Qubit to confirm its suitability for sequencing. The library was combined with the Revio polymerase kit prior to sequencing to enable binding of the primer and polymerase. PCR products were purified with cleanup beads before being sequenced on a PacBio Revio platform. The sequencing depth was assessed by examining the rarefaction curve, which leveled off at higher sequencing depths, indicating that increasing the number of sequences would result in negligible gains in species detection. The database construction and sequencing were outsourced to Beijing Baimaike Biotechnology Co., Ltd. (Beijing, China).

### 2.3. Data Analysis

CCS sequences were identified using the Lima (version 1.7.0) with barcode-based detection. The raw CCS sequence data were filtered to remove primer sequences and perform length filtering using Cutadapt (version 1.9.1). This process yielded clean CCS sequences that were devoid of primer residues. Chimera sequences were identified and removed using UCHIME (version 4.2), resulting in effective CCS sequences. These sequences were clustered at a 97% similarity threshold using Usearch (version 10), resulting in operational taxonomic units (OTUs) [32]. The SILVA reference database was used for taxonomic classification, employing the naive Bayes classifier in combination with the comparison method to assign taxonomic annotations to the featured sequences. Species classification data were obtained for each feature, and the sample community composition was analyzed at various taxonomic levels. Species abundance tables were generated at different classification levels using QIIME2 (version 2020.6). Community structure maps at different taxonomic levels were visualized using R language tools. The alpha diversity index at the OTU level was evaluated using QIIME2 (version 2020.6), and differences in alpha diversity between treatments were compared using independent-sample *t*-test. Principal coordinate analysis (PCoA) [33] and non-metric multidimensional scaling (NMDS) [34] were performed using the binary Jaccard algorithm, followed by statistical analysis using ANOSIM. Functional prediction of microbial communities was based on the KEGG database (level 3) using PICRUSt2 [35]. Statistical analysis of the experimental data was conducted using SPSS (version 20.0, IBM, Armonk, NY, USA), image processing was carried out using Adobe Photoshop (2022 version, Adobe Systems Incorporated, San Jose, CA, USA), and visualizations were generated using Excel (2021 version, Microsoft, Redmond, DC, USA).

## 3. Results

### 3.1. The Life Cycle of Chelonus formosanus

**Figure 1:**
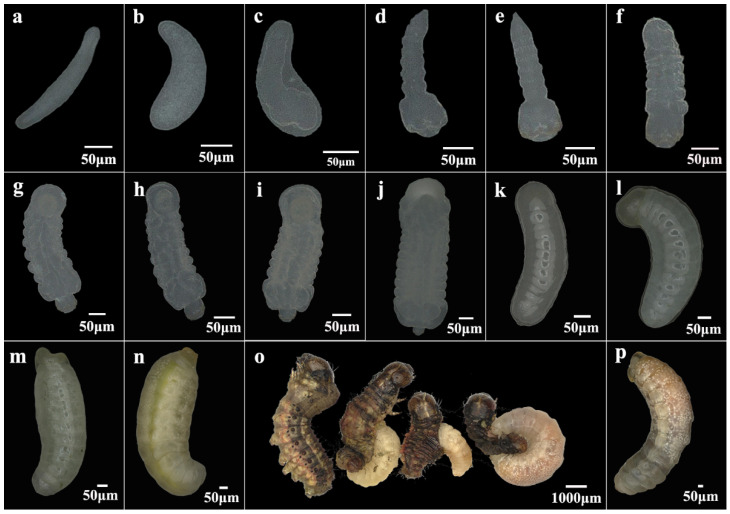
Egg-to-larval development of *Chelonus formosanus*. (**a**) Eggs dissected from the ovaries of newly emerged *C. formosanus* females. (**b**) Newly laid eggs in host hemocoel by *C. formosanus*. (**c**) Eggs shortly before hatching. (**d**–**n**) Larvae from days 1 to 11 post-parasitism. (**o**) The process by which the larvae of *C. formosanus* crawl out of *S. frugiperda* larva. (**p**) *C. formosanus* larvae fully detached from the host.

Egg to Larvae: The eggs dissected from ovaries of newly emerged female wasps are predominantly elongated and oval in shape, with a length of 252.80 ± 25.14 μm and a width of 34.56 ± 5.62 μm. Upon oviposition, these eggs undergo noticeable morphological changes, becoming shorter and wider, with a length of 180.49 ± 2.17 μm and a width of 64.59 ± 1.43 μm. Most of the eggs hatch within 24 h. After hatching, the body of wasp larvae immediately forms distinct segmentations, with a noticeably broader head compared to the abdomen and the posterior becoming more pointed. Within four to seven days of parasitism, the larvae lengthen, and the head width becomes considerably smaller relative to the thorax and abdomen. By days 8–11, larval segmentation becomes less distinct, the head nearly disappears, and the anal vesicle gradually diminishes in size. Concurrently, the body color of the larvae turns yellow. On day 11 post-parasitism, the digestive tract becomes visible with a clearly light green color, and on day 12, mature larvae emerge from the host. At this stage, white fat globules are visible, and the mouthparts and ocelli are discernible on the head (Figure 1).

**Figure 2:**
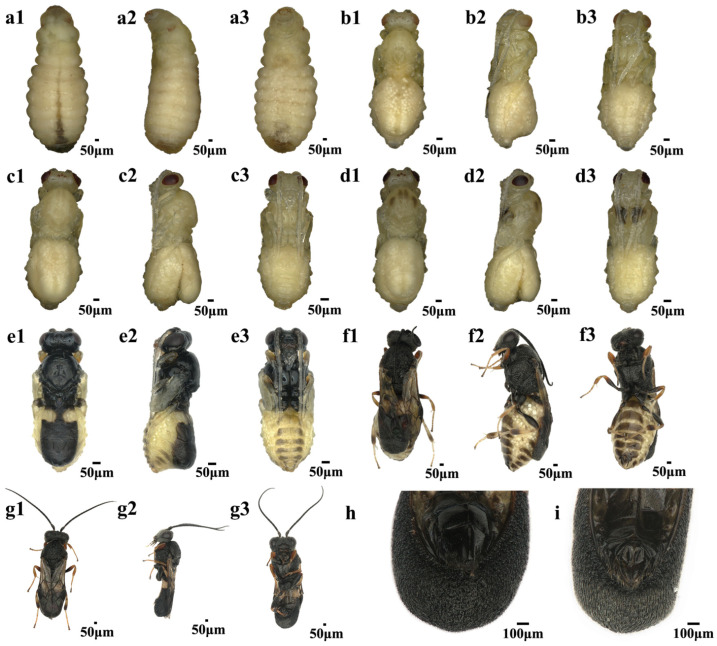
Developmental stages of *Chelonus formosanus* from pupa to adult. (**a**–**f**) *C. formosanus* pupal stages from day 1 to day 6. (**g**) Newly emerged *C. formosanus* adult. (**h**) The female genitalia of an adult wasp. (**i**) The male genitalia of an adult wasp. The orientations of images are as follows: 1, 2, and 3 represent frontal, lateral, and dorsal views at each developmental stage, respectively.

Pupa to Adult: Before pupation, *C. formosanus* larvae expel silk to construct a white cocoon, a process that lasts approximately one day. During the pupal stage, both the internal organs and external morphology of parasitoids undergo significant changes. On the second day of pupation, the differentiation of various organs begins. Between day three and day four, the compound eyes and ocelli gradually darken in color. By day five and day six, the body of *C. formosanus* gradually turns black, and the wings start to take shape. At this point, the pupal development is complete, and *C. formosanus* is about to eclose. On the 19th day post-parasitization, adult *C. formosanus* begin to emerge, mostly within one day. After eclosion, male and female wasps can be distinguished primarily by their genitalia (Figure 2).

**Figure 3:**
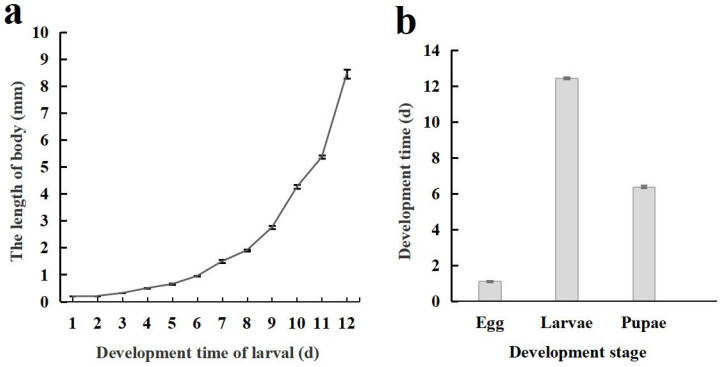
Body length changes and developmental stages of *Chelonus formosanus* larvae. (**a**) Accumulative changes in larval body length of *C. formosanus*. (**b**) Developmental stages of *C. formosanus.*

The life cycle of *C. formosanu* comprises four distinct stages: egg, larva, pupa, and adult. The body length of larvae increases in a curvilinear fashion with its development, and a notably faster growth rate was observed during later stages (Figure 3a). The duration of each developmental stage is illustrated in Figure 3b, with the total time required for development from egg to adult being 19.62 ± 0.07 days.

### 3.2. Bacterial Composition and Diversity in Chelonus formosanus

#### 3.2.1. Overview of 16S rDNA Sequencing Associated with Taxonomic Annotation

A total of 126,235 raw reads were generated by 16S rDNA high-throughput sequencing across all samples. After filtering and de-chimerization, 107,412 valid reads remained, with individual sample valid read counts ranging from 6885 to 11,286 and an average sequence length of 1459 bp. The effective range of 72.42–90.74% represents the proportion of high-quality data in the Raw CCS dataset after filtering and optimization, indicating the sequencing data’s reliability. The sequencing coverage for all samples surpassed 99.3%, ensuring that the majority of the bacterial species present at different developmental stages of *C*. *formosanus* coccinellid wasps were captured. Clustering the reads at a 97% similarity level resulted in the identification of 721 identified OTUs (Table 1).

A total of 404 bacterial species spanning 182 families and 308 genera were annotated across various taxonomic levels at different developmental stages of *C. formosanus*. Notably, the bacterial compositions of male wasps differed significantly from those of female wasps, larvae, and pupae at all taxonomic levels (*p* < 0.05). In contrast, the bacterial composition of larvae differed significantly from that of female wasps only at the genus level, with no significant differences observed at other taxonomic levels *(p* > 0.05) (Table 2).

#### 3.2.2. Composition of Bacterial Communities at Different Developmental Stages of *Chelonus formosanus*

**Figure 4:**
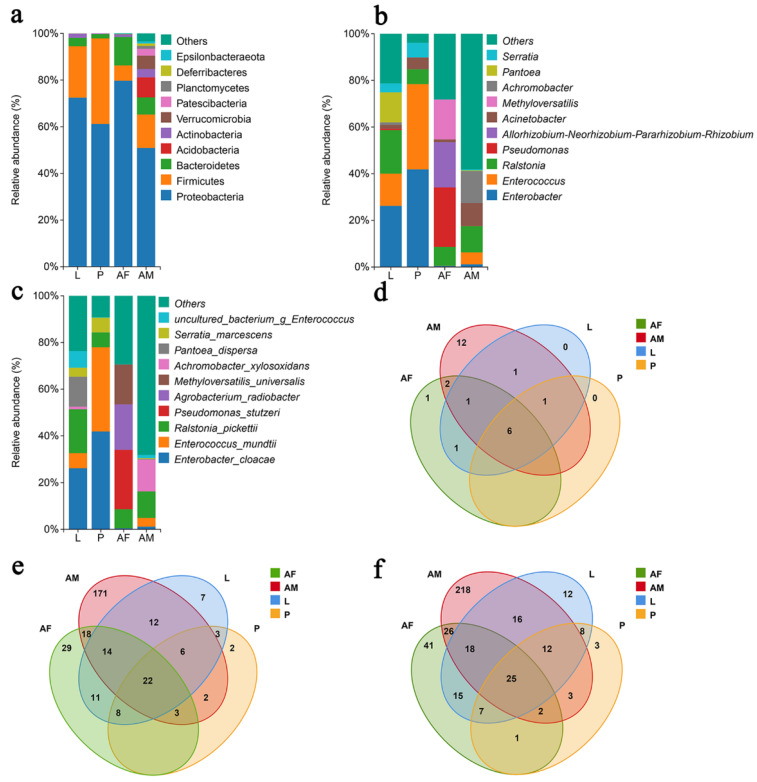
Panels (**a**–**c**) depict the relative abundance of bacteria at the phylum, genus, and species levels, respectively, across different developmental stages of *Chelonus formosanus*. Panels (**d**–**f**) show the Venn diagrams illustrating the overlap of bacterial taxa at the phylum, genus, and species levels at the same stages.

Proteobacteria is the dominant bacterial phylum across the different developmental stages of *C. formosanus*. The relative abundance of Proteobacteria in larvae, pupae, females, and males was 72.47%, 61.16%, 79.73%, and 50.91%, respectively, followed by Firmicutes and Bacteroidetes as the second most prevalent taxa (Figure 4a). At the genus level, bacterial composition and relative abundance varied considerably across developmental stages. In larvae and pupae, the predominant genera are *Enterobacter* and *Enterococcus*, while in male wasps, *Achromobacter*, *Ralstonia*, and *Achromobacter* were the most abundant genera with relative abundances of 13.70%, 13.70%, 11.30%, and 9.75%, respectively. Female wasps, on the other hand, are dominated by *Pseudomonas*, *Allorhizobium-Neorhizobium-Pararhizobium-Rhizobium*, and *Methyloversatilis*, with relative abundances of 25.48%, 19.41%, and 17.09%, respectively (Figure 4b). At the species level, *Enterobacter cloacae*, *Ralstonia pickettii*, and *Pantoea dispersa* are the dominant species in larvae, with relative abundances of 26.18%, 18.67%, and 12.86%, although *P. dispersa* was only identified in one sample. In pupae, *E*. *cloacae* and *Enterococcus mundtii* dominated, with relative abundances of 41.83% and 36.09%, while adult females exhibited a dominance of *Pseudomonas stutzeri, Agrobacterium radiobacter*, and *Methyloversatilis universalis,* with relative abundances of 25.46%, 19.41%, and 17.08%. Male wasps are primarily dominated by *Ralstonia pickettii* and *Achromobacter xylosoxidans,* with relative abundances of 11.30% and 13.63% (Figure 4c). Overall, the composition of the bacterial community in *C. formosanus* exhibited a dynamic change across developmental stages. The comparison between commonly colonized and stage-specific species revealed that 25 species are shared across the stages. The largest number of stage-specific species was observed in male wasps, followed by female wasps, with the pupal stage showing the fewest endemic species.

**Figure 5:**
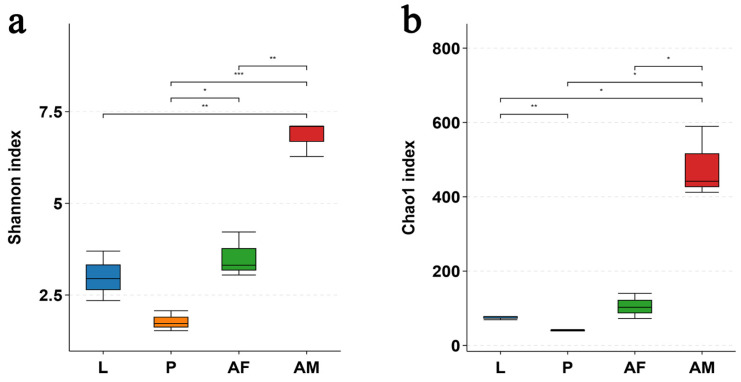
Alpha diversity of in vivo bacteria at the OTU level at different developmental stages of *Chelonus formosanus*. (**a**) Shannon index and (**b**) Chao1 index. Statistical analyses were performed using independent-sample Student’s *t*-test. Asterisks indicate significance levels: * *p* < 0.05, ** *p* < 0.01, and *** *p* < 0.001.

The bacterial diversity, as assessed by the Shannon index, and the bacterial richness, as measured by the Chao1 index, were significantly higher in male *C. formosanus* compared to larvae, pupae, and females. Among these developmental stages, pupae exhibited the lowest bacterial diversity and richness (Figure 5).

**Figure 6:**
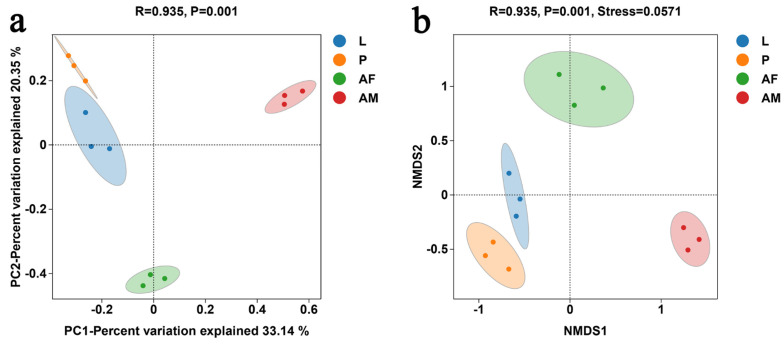
Principal coordinate analysis (PCoA) (**a**) and non-metric multidimensional scaling (NMDS) (**b**) of bacterial communities at different developmental stages of *C. formosanus*, based on the binary–Jaccard algorithm.

PCoA revealed significant differences in bacterial communities across different developmental stages of *C. formosanus* (ANOISM, R = 0.935, P = 0.001). The bacterial composition was more similar between larvae and pupae, while males were the most distinct from the other stages, indicating that males had the least similarity in bacterial species relative to other developmental stages (Figure 6a). The NMDS analysis, with a stress value below 0.2, demonstrated the reliability of the analysis (Figure 6b). The NMDS results were consistent with that of PCoA, further confirming that bacterial communities varied across different developmental stages of *C. formosanus.*

#### 3.2.3. Functional Predictions of Bacterial Community of *Chelonus formosanus*

**Figure 7:**
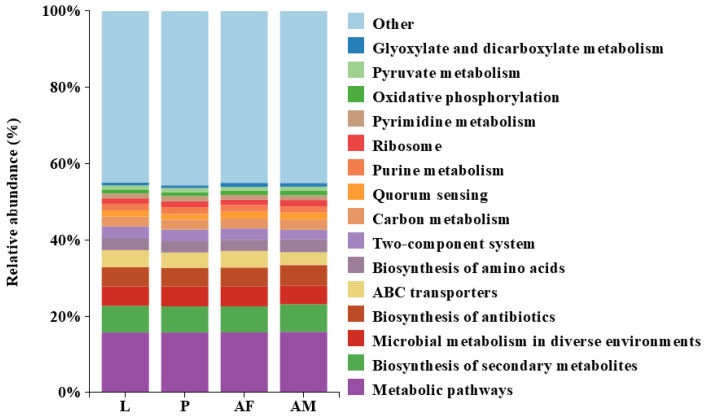
Predicted results of the Top 15 bacterial functional abundances at different developmental stages of *Chelonus formosanus*.

Bacterial profiling of *C. formosanus* revealed a high degree of similarity in the functional abundance across different developmental stages. Notably, metabolic pathways were the most abundant, highlighting their critical role in the insect’s physiological processes and their potential impact on host development and health. Additionally, the biosynthesis of antibiotics and quorum sensing were present in significant proportions at all stages, which may indicate bacterial defense mechanisms and communication. The biosynthesis of amino acids and ribosome functions were also consistently represented, emphasizing the importance of protein synthesis. In contrast, the abundance of glyoxylate and dicarboxylate metabolism, as well as pyruvate metabolism, was relatively low, suggesting that these pathways may not be central to the metabolic activities during *C. formosanus* development (Figure 7).

## 4. Discussion

*C. formosanus* is an egg-larval parasitoid wasp that includes egg, larva, pupa, and adult developmental stages. Its eggs and larvae primarily grow and develop within the host. In the present study, we observed that the parasitic capacity of *C. formosanus* ranged from 1 to 15 eggs per *S. frugiperda* egg and 6.48 ± 0.62 eggs on average. However, only one parasitoid successfully survived on the third day of post-parasitism, with occasional instances of two larvae surviving. Whether the cause of the survived parasitoid larvae killing others is by competitive interactions or by host immunity remains unclear and warrants further investigation. Previous studies have shown that *Cotesia plutellae* typically develop into wasps with mostly one individual, even if they lay multiple eggs [36]. Female wasps of mono-parasitic species engage in self-regulating parasitism when intra-specific competition is intense, thereby increasing the chances of their offspring’s survival in competition with those of other females [37]. However, only one parasitoid larva typically completes its development within each host, with excess larvae being eliminated during the physical or physiological competition [38]. Consequently, the ratio of parasitoid wasps to hosts must be carefully controlled to minimize hyperparasitism and prevent resource waste when utilizing *C. formosanus* for the biological control of *S. frugiperda* in the field.

*C. formosanus* eggs typically hatch into larvae within approximately one day. In contrast, *Psyttalia incisi* eggs take 40 to 48 h to hatch at 25 ± 1 °C [39], while *Cotesia ruficrus* eggs develop into larvae within about three days at 26 °C [40]. These findings indicate significant interspecific variation in the egg developmental duration among parasitoid wasp species. When *C. formosanus* parasitizes the host *S. frugiperda*, completing a generation takes approximately 19.62 days. However, when *Spodoptera exigua* served as the host, the development period of this wasp extends to 21 days at 27 °C [41]. This suggests that distinct host species can significantly influence the development rate of *C. formosanus.* Regarding adult emergence, *C. formosanus* exhibits diurnal eclosion, with males generally emerging earlier than females. In comparison, *P. incisi* adults emerge over a 24 h period [39]. *Zele chlorophthalmus* adults, both male and female, predominantly emerge between 06:00 and 14:00, with fewer individuals emerging between 14:00 and 22:00. Males tend to emerge 2–3 days earlier than females [42]. In *Cotesia marginiventris,* there is a clear sexual dimorphism in eclosion timing, with males emerging primarily in the morning (comprising over 90% of the total emergence), while females predominantly emerge in the evening (approximately 75%), between 20:30 and 01:30. Males typically emerge about 12 h earlier than females [43].

A total of 404 bacterial species were identified and annotated across various developmental stages of *C. formosanus*. At the phylum level, Proteobacteria was the predominant group, followed by Firmicutes in larvae, pupae, and both male and female adults. This finding is consistent with previous studies reporting that *Proteobacteria* and *Firmicutes* are the dominant bacterial phyla in the microbiota of parasitoid wasps [44]. Similar dominance of Proteobacteria has been observed in the gut microbiota of other parasitoid wasps, including *Aphelinus mali* [45], *Lepidoptoea boulardi*, *Leptopilina heterotoma* [46]**,** and *Cotesia vestalis* [47]. Furthermore, Proteobacteria has also been identified as the predominant bacterial flora in adults of other insect species, such as *Laodelphax striatellus* [48], *Propylaea japonica* [49], and *Tessaratoma papillosa* [50]. In larvae and pupae of *C. formosanus*, the dominant bacterial genera were *Enterobacter* and *Enterococcus*. Previous studies have similarly reported that *Enterococcus* is the predominant genus of *C. vestalis* larvae [47], suggesting its commonly dominant taxon in parasitoid larvae within the family of coccinellidae. Among the dominant bacteria, *E. cloacae* were found in high abundance in both the larva and pupa of *C. formosanus*. Prior research has shown that *E. cloacae* can promote the growth and development of *B. dorsalis* larvae [9], produce insecticidal proteins toxic to pests, and aggravate host insect mortality [51]. Additionally, *E. mundtii*, the dominant bacterium of *C. formosanus* pupae, has been shown to confer host resistance to entomopathogens, although it may result in reduced fecundity in *Tribolium castaneum* following exposure to *E. mundtii* isolates [52]. Moreover, the injection of *E. mundtii* into corn has been reported to significantly promote the growth of early-stage peach borer larvae with increasing length and weight [53]. The bacterium *Pseudomonas*, dominant in female *C. formosanus*, has a potential for pest biocontrol [54]. Studies showed that the gut bacterium of *Hypothenemus hampei*, *Pseudomonas,* relies on caffeine as a source of carbon and nitrogen sources to survive [55]. Another dominant bacteria in female wasps, *P. stutzeri*, can degrade chlorpyrifos [56]. In males, the dominant bacterium, *Acinetobacter*, is capable of degrading toxic phenolic glycosides secreted by host plants [57]. Furthermore, *R. pickettii*, another highly abundant and dominant bacterium found at various developmental stages of *C. formosanus*, is a versatile pathogen that can inhabit diverse environments, including aquatic habitats, sediments, plants, and drinking water [58]. These results indicate that distinct bacterial groups dominate at different developmental stages of *C. formosanus*, with each group potentially playing a critical role at specific development stages. However, further research is required to investigate their specific functions.

In this study, we revealed that the bacterial abundance was significantly lower in *C. formosanus* larvae compared to those in male and female adults, consistent with the findings reported in *Nasonia* [59]. In contrast, the intestine microbial diversity of *L. boulardi* and *L. heterotoma* larvae was markedly higher than their counterparts in adult females [46]. This disparity is attributed to their microbial communities heavily influenced by the host’s gut microbiota. Previous studies have shown that larval parasitoids depend on host gut microbes for nutrition and survival [60]. For example, the microbial communities in three *Nasonia* larvae closely resembled those of their hosts, suggesting that larvae acquire the predominant microbial taxa directly from their hosts [59]. Furthermore, significant differences in bacterial composition were observed between male and female *C. formosanus* adults, with male wasps harboring a higher number of bacterial species than females. This pattern is consistent with observations in other insect species, such as *Chrysoperla sinica* [61], *Leptocybe invasa* [62], and *Helicoverpa armigera* [63], where males also exhibited higher microbial diversity. Conversely, *P. japonica* females had greater gut bacterial abundance and diversity than males, while in *Ericerus pela*, females displayed higher bacterial diversity than males, likely due to the fact that male wasps develop under a protective ash layer and experience a more simplified environment compared to females [64]. These findings suggest that variations in the bacterial communities between wasp male and female adults may be related to the differences in their physiological metabolism and life history traits. Picrust2-based functional prediction of bacterial KEGG pathways at different developmental stages of *C. formosanus* revealed that metabolic pathways were the most abundant functional category across all different development stages of *C. formosanus*. The presence of genes associated with metabolic pathways is likely crucial for nutrient acquisition and host growth [28]. The biosynthesis of secondary metabolites was also prominently represented. Although the abundance of bacterial functional categories appeared similar across developmental stages, given that the functional predictions from PICRUSt2 are somewhat speculative, it is necessary to further verify through metagenomic studies whether these functions truly exist and play specific roles in the growth and development of *C. formosanus*.

This study represents the first comprehensive examination of developmental dynamics in *C. formosanus*. However, the interspecies competition between multiple individuals within the host and the underlying mechanism of the eventual sole surviving wasp remains to be further elucidated. Additionally, the species composition and abundance of intestinal bacteria were unveiled across different developmental stages of *C. formosanus*, thereby broadening the existing knowledge of its resided microbiota. These findings provide valuable insights into the core microorganisms in *C. formosanus* and shed light on their potential biological functions, which may serve as a foundation for exploring their application for pest control.

## 5. Conclusions

In summary, this study provides a comprehensive analysis of the developmental and morphological dynamics of *Chelonus formosanus*, establishing a morphological foundation on this wasp and enabling deeper investigations. The dominant bacterial taxa were characterized at different developmental stages, revealing significant differences in bacterial composition and abundance across developmental stages, as well as between female and male adults. Notably, males harbored the greatest number of bacterial species and endemics, while pupae exhibited the lowest bacterial abundance and diversity. Additionally, the functional profiling of the most abundant category of bacterial communities was annotated as metabolic pathways across various developmental stages. However, the specific physiological roles of these microbial functions warrant further investigation.

## Figures and Tables

**Table 1:** The 16S RNA sequencing data of in vivo bacteria at different developmental stages of *Chelonus formosanus.*

Sample	Raw CCS	Clean CCS	Effective CCS	AvgLen (bp)	Effective (%)	OTU	Coverage (%)
L1	10,954	9850	9691	1464	88.47	73	99.88
L2	9174	7951	7853	1465	85.60	61	99.84
L3	8280	7482	7429	1461	89.72	69	99.80
P1	13,014	11,525	11,212	1468	86.15	38	99.93
P2	11,513	10,309	10,093	1468	87.67	34	99.91
P3	10,616	9601	9531	1476	89.78	37	99.95
AF1	8012	7308	7270	1447	90.74	96	99.83
AF2	13,033	11,529	11,286	1446	86.60	71	99.97
AF3	10,667	9348	9094	1446	85.25	136	99.92
AM1	10,000	8256	7832	1457	78.32	578	99.35
AM2	12,753	9809	9236	1458	72.42	420	99.58
AM3	8219	7216	6885	1457	83.77	405	99.73

Note: L, P, AF, and AM stand for larvae, pupae, adult females, and adult males of *Chelonus formosanus*, and 1, 2, and 3 stand for three replications, respectively. The same is below.

**Table 2:** Annotated taxonomic units at different stages of *Chelonus formosanus.*

Sample	Phylum	Class	Order	Family	Genus	Species
L	5.00 ± 0.58 b	8.00 ± 0.00 b	16.00 ± 1.15 b	21.00 ± 1.53 b	28.00 ± 1.00 c	36.00 ± 1.15 b
P	6.33 ± 0.33 b	10.00 ± 0.57 b	22.67 ± 0.88 b	36.33 ± 3.06 b	50.67 ± 0.88 bc	66.33 ± 3.48 b
AF	7.33 ± 1.20 b	11.33 ± 0.88 b	24.00 ± 1.00 b	40.00 ± 2.65 b	63.00 ± 6.03 b	75.33 ± 7.67 b
AM	21.33 ± 0.88 a	51.00 ± 3.60 a	95.00 ± 6.56 a	140.00 ± 19.97 a	210.00 ± 31.75 a	266.67 ± 25.33 a
Total	25	61	116	182	308	404

Note: Different lowercase letters indicate significant differences (*p* < 0.05) (Duncan’s new complex polarity method).

## Data Availability

Raw sequencing data were deposited in the NCBI Short Read Archive (SRA) BioProject PRJNA1206106.
